# Depression and Its Associated Factor Among Women Using Hormonal Contraceptives: A Cross-Sectional Study in Jimma Town Public Health Facilities, Southwest Ethiopia, 2022

**DOI:** 10.1089/whr.2024.0100

**Published:** 2025-03-25

**Authors:** Beniam Worku, Nafyad Tolosa

**Affiliations:** School of Medicine, College of Health Science, Arsi University, Asella, Ethiopia.

**Keywords:** depression, hormonal contraceptives, Beck Depression Inventory II

## Abstract

**Background:**

Depression is a significant mental disorder that affects >350 million individuals globally. It is noteworthy that females are approximately twice as likely as males to experience depression, with the incidence of depression in females rising during early adolescence. The fluctuation in levels of gonadal hormones contributes to the increased occurrence of depression in females. The use of hormonal contraceptives suppresses the natural production of these hormones, which, in turn, raises the risk of developing depressive symptoms in women. The complex relationship between fluctuating hormones and depression in women is multifaceted, with both natural hormonal changes and hormonal contraceptive use potentially impacting emotional well-being and susceptibility to affective disorders.

**Methods:**

An institution-based cross-sectional study was conducted on a sample of 423 individuals. A simple random sampling technique was used for participant selection. The Beck Depression Inventory II screening tool, along with pretested structured interview questionnaires, was used to collect sociodemographic information as well as data on the use of hormonal contraceptives and menstrual history. Trained nurses administered the questionnaires and measured the body mass index (BMI) using standard measuring tools. The collected data were entered into Epi-Data Manager version 4.4.1 and then exported to Statistical Package for Social Sciences version 26 for statistical analysis. Bivariate and multivariate logistic regression analyses were performed to determine the association between dependent and independent variables. All explanatory variables with a *p*-value <0.25 in bivariate analysis were entered into the multivariable logistic regression model. A *p*-value <0.05 in the multivariate logistic regression analysis was used to determine statistically significant variables.

**Results:**

The prevalence of depression among the study participants was 38.8%. The age category 18–25 years, poor social support, uncomfortable marital relationship, injectable hormonal contraceptives, and BMI status >25 kg/m^2^ were variables that became significantly associated with depression in this study.

**Conclusions:**

This study has shown that the prevalence of depression among contraceptive users was 38.8%. The age-group of 18–25 years, poor social support, an uncomfortable marital relationship, use of injectable hormonal contraceptives, and a BMI status >25 kg/m^2^ have an impact on the prevalence of depression. Based on these findings, it is recommended that women’s health initiatives prioritize mental health services. Providing adequate mental health support and counseling can help address the prevalence of depression among contraceptive users. It is also suggested that health care providers carefully evaluate the risks and benefits for women before initiating hormonal contraception. It is important to provide special support to women who have poor social support, conflicts with their husbands, are overweight or obese, and are in their early twenties. To build on this knowledge, further prospective studies are suggested to explore the relationship between hormonal contraception and depression.

## Background

Depression is a mental health disease that is characterized by prolonged feelings of sadness or despair, as well as a lack of interest in activities.^1^ It is associated with symptoms such as low mood, diminished pleasure or interest, reduced energy levels, feelings of guilt or low self-esteem, disrupted sleep or appetite patterns, and difficulty concentrating.^2^

The symptoms of depression are influenced by various brain regions. The neocortex and hippocampus play a role in cognitive aspects of depression, such as memory impairments, suicidal thoughts, and feelings of worthlessness, hopelessness, remorse, and dread.^3^ The ventral striatum or nucleus accumbens, amygdala, and related brain areas, including the striatum, are involved in emotional memory and may contribute to anhedonia (reduced pleasure in activities), anxiety, and decreased motivation commonly observed in depressed individuals.^4^ Additionally, the hypothalamus is responsible for regulating neurovegetative symptoms of depression, including disturbances in sleep patterns, appetite changes, energy levels, and a decline in interest in sexual and pleasurable activities.

Depression is the major mental disorder that has a high prevalence rate.^5^ It is the most severe disorder that causes morbidity and mortality all over the world, and this disorder leads to other problems and affects an individual’s life. The World Health Organization 2017 reported that 350 million people are living with depression.^6^ Depression is the fourth most important contributor to the global burden of disease.^7^ Depression is also the major contributor to suicide deaths, which number close to 800, 000 per year. There is now widespread recognition of the significant burden that depression imposes on individuals and their careers, health services, and communities throughout the world.^8^ It is the most common mental disorder in community settings, starts at a young age, and is a major cause of disability across the world.^3^

Depression has a more significant negative impact on one’s overall health compared to other major non-communicable diseases.^9^ People with depression not only feel emotional pain, but it also affects their social life, work, physical health, and can even lead to death. Depression makes it hard for people to work well, causing financial problems for them and their families.^10^ In severe cases, depression can lead to suicide, making up nearly 1% of all deaths. It is alarming that about two-thirds of these suicides are by people who are depressed, leading to almost 1 million deaths each year, which means around 3,000 people die by suicide every day.^11^

It is widely reported that there is a sex disparity in the prevalence of depression, with women being nearly twice as likely to develop depression compared with men.^4^ Surveys conducted among children under the age of 15 show no significant difference in depression rates between boys and girls. However, after puberty, the prevalence of depression becomes twice as high among females compared to males.^12^ This raises important questions about the role of sex hormones in the higher prevalence of depression among women.

The relationship between fluctuating hormones and depression in women is complex and multifaceted. Research indicates that hormonal changes, such as those during the perimenstrual phase, can contribute to emotional disturbances and sleep disruptions, potentially increasing the risk of affective disorders.^13^ On the contrary, the initiation of hormonal contraception has been linked to a higher risk of developing postpartum depression in women who are sensitive to hormonal transitions, suggesting that suppressing these fluctuations can also play a role in the incidence of depression.^14^ Therefore, while natural hormonal fluctuations can impact emotional well-being, the use of hormonal contraceptives, by altering these fluctuations, may unmask susceptibility to depression, highlighting the intricate interplay between hormones and mental health in women.

Many women report changes in mood associated with hormonal fluctuations during experiences such as the premenstrual period, menopause, use of hormonal contraceptives, and hormone replacement therapy.^4^ The use of hormonal contraceptives, for instance, can modify the balance of endogenous progesterone and neurosteroids, leading to reduced levels of testosterone and dehydroepiandrosterone, as well as increased levels of sex hormone binding globulin. These changes in hormone levels might increase vulnerability to depression in women.^15^

The addition of progesterone to hormone therapy has been found to induce adverse mood effects in women. One likely mechanism is the action of progesterone metabolites on the γ-amino butyric acid a receptor complex, which is the major inhibitory system in the human central nervous system. During the luteal phase of the menstrual cycle in fertile women, levels of neuroactive metabolites of progesterone increase, and some women experience negative mood symptoms.^16^ Additionally, external progestins, more so than natural progesterone, have been shown to increase levels of monoamine oxidase, which degrades serotonin concentrations and potentially leads to depression.^16^

Contraceptive associated with increased risk of depression according to studies done in different part of the world. For example, study conducted in Saudi Arabia showed that prevalence of depression is 55% among women using hormonal contraceptives.^17^ Cohort study done on women using hormonal contraceptive in Denmark showed notably higher relative risk of first use of antidepressants and first diagnosis of depression when compared with non-users.^18^ The impact of age on the effects of contraceptives on female mood is a crucial factor to consider. Research indicates that women who begin using oral contraceptives at or before the age of 20 may face a higher risk of depression during the first two years of use, with the risk decreasing over time.^19^ Additionally, adolescents using hormonal contraceptives have shown higher levels of individual depressive symptoms such as sadness, reduced libido, feelings of pessimism, and failure.^20^

The use of hormonal contraceptives has been associated with negative mood changes and depression in some women, but studies have yielded conflicting results. Some studies have reported increased negative mood changes,^21–23^ while others have failed to find any significant impact^24,25^ and others found negative associations.^26,27^ These inconsistencies between the different studies may be due to variations in the age of participants and non-evaluation of social circumstances. Therefore, this study aims to estimate the prevalence of depression among hormonal contraceptive users who live in Jimma town Ethiopia and to identify factors that may predispose hormonal contraceptives users to depression.

The findings of this study will provide knowledge for health care professionals working on planning services for evidence-based practice. In addition, this study will reveal important factors that are related to depression among women; therefore, Ministry of Health and nongovernmental organizations that are working on women health can benefit from the identified factors in this study.

It is also hoped that the study will benefit researchers who are interested in conducting further study on the related topics.

## Methods

### Study setting

From April to November 2022, the study was carried out in six public health facilities in Jimma Town. The town is 352 km southwest of the capital Addis Abeba and has an estimated population of 207,573, making it one of the most populous cities in the Oromia regional states.^28^ The town has about 30 private and four nonprofit medium clinics in addition to one zonal, one medical center, and four public health centers. The two public hospitals were Shenen Gibe Zonal Hospital and Jimma Medical Center, which offer more of curative treatments to populations in Oromia and the Southwest of Ethiopia.

### Study design and period

Institution-based cross-sectional study was conducted from October 3 to November 13, 2022.

### Source population

The population included all women who visited family planning service in the health facilities.

### Study population

The population included women using hormonal contraception and those who fulfill the inclusion criteria for actual participation in the study process.

### Inclusion criteria

These criteria included women aged between 18 and 45 years and clinically stable women.

### Exclusion criteria

The criteria included chronic alcohol and/or tobacco use and chat chewing, breastfeeding (≤6 months postpartum), and the use of other medication, known to have antidepressant affects.

### Sample size determination and sampling technique

Sample size was determined by using a single population proportion formula with a prevalence of 50% (*p* = 0.5) since there is no finding that indicates prevalence in previously done research on the same study population or the same area, 95% confidence interval (CI), and margin error of 5% (w = 0.05).

$$n=\frac{\left(z\alpha /2)2\;(1-p\right)}{d^{2}}$$

where

n = (1.96)2 * 0.5(0.5)/0.052 n =384

By adding 10% non-response rate, the minimum final sample size of the study was 423.

The total sample size was distributed to each health facilities based on probability proportional to size allocation method. Finally, each individual is selected by simple random sampling.

### Data collection procedures

Four trained nurses collected the data. All women were screened for eligibility based on a medical interview. Study participants answered questions in the questionnaire, which were relevant to their sociodemographic information, hormonal contraceptive use, and other factors. Afterward, blood pressure, height, and body weight were measured for each participant. Body mass index (BMI) was calculated by dividing weight (kilograms) by the square of height (square meters).

Data were collected using a pretested structured interviewer-administered questionnaire. The questionnaire included sociodemographic characteristics, hormonal contraceptive use (type and duration of use), and history of menstrual cycle (i.e., whether they were in the premenstrual or postmenstrual period) and social support questionnaires. The Beck Depression Inventory (BDI) II screening questionnaire was used to screen for depression and to grade its severity. The BDI is the most widely used screening method for depression and has a high degree of sensitivity and specificity for detecting depression. This screening tool is also being used in Ethiopia both in research and clinical practice to detect depression among the general population. The scale’s format is clear, simple to administer, and easily understood by the population and can be used to detect depression in normal populations regardless of age or sex. A number of studies have established the validity and reliability of the BDI-II in different populations and settings. The content validity of the scale has been improved by rewording and adding items to assess *Diagnostic and Statistical Manual of Mental Disorders*, Fourth Edition (DSM-IV) criteria for depression. The measure has been evaluated for convergent and divergent validity in different studies and yields a positive correlation with the Center for Epidemiological Studies Depression Scale (r = 0.69), Coolidge Axis II Inventory Depression subscale (r = 0.66), and Hamilton Rating Scale for Depression (r = 0.66), which supports the validity of the measure. Regarding the reliability of the measure, the internal consistency reliability was high in the original manual with a Cronbach’s α of 0.92 for the outpatient population. The BDI contains 21 items that assess cognitive, behavioral, affective, and somatic components of depression. The clinical observations were consolidated systematically into 21 symptoms and attitudes that could be rated from 0 to 3 in terms of intensity. The 21-item is scored on a scale of 0–3 in a list of four statements arranged in increasing severity about a particular symptom of depression, bringing the BDI-II into alignment with DSM-IV criteria. Total BDI scores range from 0 to 63. Scoring was conducted as follows: normal (0–13), mild depression,^14–18^ moderate depression,^19–27^ and severe depression.^28–52^ Amharic and Afan Oromo language questionnaire versions were used for data collection.

### Ethical consideration

Ethical clearance was obtained from the institutional review board of Jimma University Health Institute. Following the approval of the official letter, it was written to concerned bodies. The nature, purpose, and benefits of the study were explained to the study participants. Participants’ rights to refuse or discontinue participation at any time they want were strictly respected. To preserve the confidentiality, the recorded data were accessed by only four nurses who collected the data and principal investigator. The same four trained nurses collected data across all facilities ensuring consistency in data collection methods and maintaining confidentiality. All data were securely stored and handled in accordance with ethical guidelines.

### Analysis processing and analysis

The data were coded, edited, and entered into Epi data version 4.1 and exported to Statistical Package for Social Sciences version 26 for analysis. Descriptive analysis such as frequency distribution and cross tabulation was performed. The outcome and independent variables were entered into a binary logistic regression one by one, in order to explore each independent variable association with outcome variable. Finally, multivariate logistic regression was computed for some of independent variables taken from the bivariate analysis. In this study, independent variables with *p* < 0.25 were selected as candidates for further analysis to identify factors independently associated with outcome variable in the final model. Adjusted odds ratio (AOR) with 95% CI was computed, and statistical significance was set at *p*-value of <0.05 in the final multiple logistic regression models.

### Operational definitions

#### Depression

Based on BDI-II, score depression was categorized as follows: normal (0–13), mild depression,^14–18^ moderate depression,^19–27^ and severe depression.^28–52^

#### Hormonal contraception

Hormonal contraception included birth control methods that contain estrogen and progesterone or progesterone only.^30^

#### Injectable hormonal contraception

According to this study, injectable contraception is defined as Depo Provera, which is given by intramuscular injection every 3 months.^30^

#### Oral contraception

Oral contraception included birth control pill that is taken orally once a day.^30^

#### Implant

Implant included hormonal birth control that is placed under the skin of the upper arm.^30^

#### Overweight

Women are categorized as overweight if their BMI is between 25.0 and 29.9 kg/m^2^.^31^

### Obese

Obesity is indicated by BMI ≥30.0 kg/m^2^ or more.^31^

#### Level of social support

For this study, social support is measured using the Oslo 3-item social support scale and a score of:

- 3–8 for poor support.
- 9–11 for moderate support.
- 12–14 for strong support.^32^

## Results

### Sociodemographic characteristics of respondents

Totally, 423 participants were enrolled in the study, with a 100% response rate. The majority of participants were married (370, 85.5%). More than half of the respondents (234, 55%) were between the ages of 18 and 25. Of all participants, the majority (324, 74.5%) were Oromo by ethnicity and 228 (52.7%) were Muslim by religion. A significant number of participants (314, 85.2%) had received modern education, ranging from primary to tertiary level. About 194 (44.8%) were housewives, followed by government employees (83, 19.2%). About 183 (43.3%) of respondents reported having moderate social support and 152 (59.5%) reported a monthly household income of 2,000–5,000 Ethiopian Birr (ETB), which is considered a low to medium income level in Ethiopia. Regarding their relationship with their husbands, 178 (47.8%) reported living comfortably with their husbands. The majority of respondents (194, 46.1%) had one to two children, as indicated in Table 1.

**Table 1.** Socio-demographic Characteristics of Women Using Hormonal Contraceptives in Public Health Facilities in Jimma Town, 2022 (*n* = 423)

Variables	Categories	Frequency (*n* = 423)	Percentage (%)
Age(in years)	18–25	234	55.3
	26–30	150	34.8
	31–45	42	9.9
Marital status	Married	370	87.5
	Single	36	8.5
	Divorced	10	2.4
	Widowed	7	1.7
Ethnicity	Oromo	324	76.6
	Amhara	42	9.9
	Yem	20	4.7
	Others^a^	37	8.8
Educational status	Illiterate	109	25.8
	Read and write	127	30.3
	Primary education	40	9.5
	Secondary education	86	20.3
	College and above	61	14.4
Occupation	Housewife	194	45.9
	Government employee	83	19.6
	Merchant	44	10.4
	Others^b^	132	24.1
Household monthly income	<2,000	120	28.4
	2,000–5,000	152	59.5
	>5,000	51	12.1
Number of children	None	90	21.3
	1–2	195	46.1
	>3	138	32.6
Relationship with husband	Not comfortable/at all	62	14.7
	Somewhat comfortable	132	31.2
	Comfortable/very	178	42.1
Social support	Poor support	144	34.1
	Moderate support	183	43.3
	Strong support	96	22.6

^a^ Wolayita, gamo, gurago, and so on.

^b^ Nongovernmental, private worker, jobless, and student.

### Hormonal contraceptive and menstruation-related characteristics of study participants

Out of the total study participants, 185 (43.7%) were using injectable type of hormonal contraceptive followed by implant (50, 35.5%). Regarding menstruation, among those in regular menstrual cycle (i.e., 166 of the respondents are amenorrheic in this study), more than half of the respondents (153, 59.5%) were found in the postmenstrual phase during interview time, as indicated in Table 2.

**Table 2.** Contraceptives and Menstruation-Related Characteristics of Women Using Hormonal Contraceptives in Public Health Facilities in Jimma Town, 2022 (*n* = 423)

Variables	Categories	Frequency	Percentage (%)
Type of hormonal contraceptives	Oral	88	20.8
	Injectable	185	43.7
	Implant	150	35.5
Duration of use of hormonal contraceptives	More than three months	43	10.2
	More than 6 months		
	More than a year	105	24.8
	More than 3 years	114	27.0
		161	38.1
Menstrual period	Pre	104	40.5
	Post/mens	153	59.5

### Anthropometric status of study participants

Majority of study participants’ BMI status was on normal range (258, 61%) followed by underweight (139, 32.8%). Similarly, majority of study participants’ waist-to-hip ratio was on normal range (399, 94.3%), as indicated in Table 3.

**Table 3.** Anthropometric Status of Women Using Hormonal Contraceptive in Public Health Facilities in Jimma Town, 2022 (*n* = 423)

Variables	Categories	Frequency	Percentage (%)
Body mass index (kg/m^2^)	<18.5	139	32.8
	18.5–24.9	258	61
	≥25	26	6.2
Waist-to-hip ratio (cm)	<0.85	399	94.3
	≥0.85	24	5.7

### Prevalence of depression

**FIG. 1.**
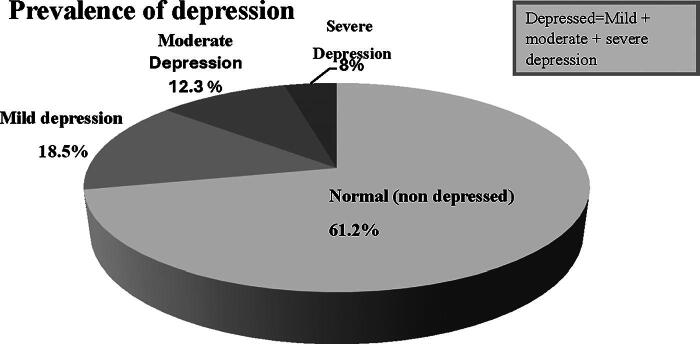
Level of depression and its prevalence among women using hormonal contraceptives in public health facilities in Jimma town, 2022.

The study participant’s depression status was assessed by BDI-II, and the prevalence of depression in the study participants was 38.8% (164). About 61.2% (259) of the participants were without depression (normal), and 18.5% had mild depression, 12.3%^51^ had moderate depression, and 8%^33^ had severe depression (Fig. 1).

### Factors associated with depression

In the bivariable logistic regression analysis, variables such as 18–25 years age category, being single, income <2,000 ETB, illiterate in educational status, an uncomfortable marital relationship, less social support, being in the premenstrual phase, BMI status ≥25 kg/m^2^, waist-to-hip ratio >0.85 cm, being a housewife, injectable type of hormonal contraceptives, and use of hormonal contraceptives more than 3 years were variables that fulfilled the minimum requirement (in this study *p*-value <0.25 level of significance) for further analysis and entered into the multivariate analysis (Table 4).

**Table 4.** Bivariate Analysis of Factors Associated with Depression Among Women Using Hormonal Contraceptives in Jimma Town, 2022

Variables	Depression		COR and 95% CI	*p*-Value
	No *n* (%)	Yes *n* (%)		
Age				
18–25	135 (52.5)	99 (60.3)	2.041 (1.036–4.026)	0.004^*^
26–30	102 (39.7)	45 (27.5)	1.70 (0.713–2.264)	0.877
31–45	20 (7.8)	20 (12.2)	1	1
Marital status				
Married	239 (92.3)	131 (79.9)	1	1
Single	15 (5.8)	21 (12.8)	2.071 (1.181–3.632)	0.011*
Divorced	3 (1.1)	7 (4.2)	1.226 (0.511–2.781)	0.641
Widowed	3 (1.1)	5 (3.1)	1.232 (0.590–2.571)	0.576
Educational status				
Illiterate	69 (26.6)	40 (24.4)	1.236 (0.546–2.801)	0.206*
Read and write	82 (31.7)	45 (27.3)	1.283 (0.675–2.427)	0.447
Primary school	25 (9.7)	15 (9.2)	0.935 (0.486–1.821)	0.851
Secondary school	48 (18.5)	38 (23.2)	0.31 (0.1311–0.870)	0.024
College and above	35 (13.5)	26 (15.8)	1	1
Household monthly income				
<2,000	66 (20.6)	54 (36.7)	2.99 (1.66–5.39)	0.000*
2,000–5,000	252 (78.2)	81 (55.1)	0.953 (0.398–2.273)	0.911
>5,000	4 (1.2)	12 (8.2)	1	1
Relationship with husband				
Not comfortable/at all	43 (17.9)	19 (14.4)	1.62 (0.89–3.15)	0.439
Somewhat comfortable	67 (27.9)	65 (49.2)	5.803 (3.574–9.753)	0.000*
Comfortable/very	130 (54.2)	48 (36.4)	1	1
Social support				
Poor support	78 (30.1)	96 (58.5)	4.25 (2.58–6.88)	0.000^*^
Moderate support	74 (28.6)	37 (22.6)	0.89 (0.36–2.21)	0.769
High support	107 (41.3)	31 (18.9)	1	1
Menstrual period				
Pre	40 (30)	64 (51.6)	2.485 (1.46–4.14)	0.001*
Post/menstrual	93 (70)	60 (48.4)	1	1
Type of hormonal contraceptives				
Oral	57 (22)	31 (48.4)	1	1
Implant	90 (34.7)	60 (36.5)	1.40 (0.60–3.27)	0.431
Injectable	112 (43.3)	73 (44.6)	2.27 (1.077–4.78)	0.031*
Duration of contraceptive use				
More than 3 months	27 (10.4)	16 (6.1)	1	1
More than 6 months	87 (33.6)	18 (10.1)	0.64 (0.324–1.29)	0.291
More than a year	61 (23.5)	53 (32.3)	0.68 (0.332–1.401)	0.297
More than 3 years	84 (32.5)	77 (46.5)	2.88 (1.286–6.394)	0.010*
Waist-to-hip ratio (cm)				
<0.85	241 (96.4)	158 (91.3)	1	1
≥0.85	9 (3.6)	15 (8.7)	1.433 (0.812–2.416)	0.208
BMI status (kg/m^2^)				
18.5–24.9	171 (66)	87 (53)	1	1
<18.5	81 (31.2)	58 (35.3)	1.24 (0.40, 3.76)	0.705
≥25	7 (2.8)	19 (11.7)	3.95 (2.36, 6.52)	0.000*
Hypertension	26 (10)	22 (14)	2.06 (0.90, 4.69)	0.086*
Hypertension	32 (12.5)	21 (12.8)	1.094 (0.756–1.583)	0.634

The bolded values indicate statistically significant associations.

^*^ Factors that have association at *p*-value <0.25.

BMI, body mass index, CI, confidence interval.

The result of multivariate analysis shows that depression was significantly associated with age category 18–25 years (2.307; 1.055–5.049), having poor social support (3.376; 1.506–7.570), having an uncomfortable marital relationship (2.9; 1.5, 5.4), injectable types of hormonal contraceptives (2.2; 1.07–4.7), and BMI status above 25 kg/m^2^ (2.167; 1.169–4.017).

Accordingly, the odds of having depression among the age category 18–25 years were 2 times higher (AOR = 2.3; 95% CI: 1.05, 5.04) as compared with age category of 31–45 years. The odds of having depression among clients with poor social support were 3.3 times (AOR = 3.3; 95% CI: 1.5, 7.5) higher compared with those clients who have strong social support. Similarly, clients who have a somewhat comfortable marital relationship are 3 times (AOR = 2.9; 95% CI: 1.5, 5.4) more likely to have depression compared with those who have a comfortable marital relationship. Odds of depression of those using injectable types of hormonal contraceptives were 2.2 times (AOR = 2.26; 95% CI: 1.07, 4.77). The odds having depression among women with a BMI above 25 kg/m^2^ (AOR = 2.1; 95% CI: 1.16, 4.0) were 2 times more likely to have depression than those in the normal range BMI (Table 5).

**Table 5.** Multivariate Analysis of Factors Associated with Depression Among Women Using Hormonal Contraceptives in Jimma Town, 2022

Variables	Category	No depression *n* (%)	Depression *n* (%)	Adjusted odds ratio and 95% CI	*p*-Value
Age(in years)	18–25	135 (57.8%)	99 (42.2%)	2.307 (1.055–5.049)	0.004^*^
	26–30	102 (69.3%)	45 (30.7%)	1.273 (0.682–2.377)	0.448
	31–45	20 (50%)	20 (50%)	1	
Social support	Poor support	78 (44.8%)	96 (55.2%)	3.376 (1.506–7.570)	0.003^*^
	Moderate Support	74 (66.7%)	37 (33.3%)	1.525 (0.692–3.361)	0.295
	High support	107 (77.5%)	31 (22.5%)	1	
Relationship with husband	Not comfortable/At All	43 (69.3%)	19 (30.7%)	1.170 (0.691–1.982)	0.559
	Somewhat comfortable	67 (50.8%)	65 (49.2%)	2.928 (1.570–5.463)	0.001^*^
	Comfortable/Very	130 (73%)	48 (30%)	1	
Type of hormonal contraceptives	Oral	57 (64.8%)	31 (35.2%)	1	
	Implant	90 (60%)	60 (40%)	0.816 (0.473–1.408)	0.465
	Injectable	112 (60.5%)	73 (39.5%)	1.908 (1.076–4.77)	0.045^*^
BMI status	18.5–24.9	171 (66%)	87 (53%)	1	
	<18.5	81 (31.2%)	58 (35.3%)	1.24 (0.40–3.76)	0.705
	≥25	7 (27%)	19 (73%)	2.167 (1.169–4.017)	0.003^*^

^*^ Factors that have association at *p*-value <0.05.

## Discussion

The finding of this study showed that the prevalence of depression among hormonal contraceptives in Jimma town was 38.8% (95% CI = 34.1%–43.6%), which is almost similar to the study done in Iran (37.7%).^33^ However, the finding of the present study was less than the study done among hormonal contraceptive users in Saud Arabia (55%)^17^ and Iran (47.8%).^34^ The possible reason for the difference might be the study population difference, which had different sociodemographic characteristics, sample size, and methods of data collections. Our study primarily focused on women aged 18–45 years, encompassing a broad age range that reflects a diverse cohort of hormonal contraceptive users. In contrast, the study conducted in Iran predominantly included women aged 20–35 years, indicating a narrower age range compared to our research population. Similarly, the study in Saudi Arabia concentrated on women aged 25–45 years, showcasing variations in age distribution compared with our study.

The prevalence of depression in this study is far above the previous study report from Australia (10%).^35^ The discrepancy may be because a completely different clinical rating tool was used within which two stages of assessing steps (both screening and diagnostic tools) were employed in the study. It was conducted within the method of administering General Health Questionnaire 12 (GHQ-12) initially that was followed by a semi-structured clinical interview of diagnostic manuals for mental disorders (SCID). The patient who becomes positive for GHQ-12 is interviewed with SCID, which indicates more accurate screening of depression than the present study.

Regarding factors affecting prevalence of depression, the study finding revealed that age category 18–25, poor social support, uncomfortable marital relationship, injectable type of hormonal contraceptives, and BMI status above 25 kg/m^2^ had significant association with depression.

The results of the study indicate that the age-group of 18–25 years is a significant predictor for depression among hormonal contraceptive users. This may be attributed to the long-lasting changes in brain and behavior caused by gonadal hormones during critical periods of social, cognitive, reproductive, and physiological development, such as adolescence and late adolescence. The amygdala, prefrontal cortex, and hippocampus, which are important regions of the brain related to emotions, are still maturing during this age and may be particularly sensitive to changes in sex hormones.^36–38^ One potential reason for the absence of a clear link between the duration of contraception use and depression could be the concept of hormonal equilibrium and adaptation.^20,39^ Studies have shown that while there may be an increased risk of depression during the initial years of contraceptive use, this risk tends to decline over time as the body adapts to the hormonal changes induced by the contraceptives.^19^

Another signification factor that is associated with depression is poor social support. Social support is an important environmental resource in an individual’s social life, affecting their physical and mental health and behavior patterns. Good social support allows individuals to gain self-esteem and self-efficacy more easily, thereby resisting the generation of negative emotions such as depression. When an individual is under stress, social support makes them underestimate the hazards and the varieties of stress by enhancing their perceived coping capacities. Social support can also provide problem-solving strategies to the individual, reduce the importance of the problem, and alleviate the harmful effects of stress experience. These effects can reduce the intensity of the relationship between stress and depression.^40,41^ Therefore, individuals with poor social support are more prone to having low self-esteem, negative emotional reactions to life stressors, and poor coping mechanisms, which increase the likelihood of having depression. This finding is consistent with previous studies from Saudi Arabia and other parts of the world.^17,40–43^

The study mentioned that women who had marital problems were more likely to experience depression than those who lived with their husbands comfortably. This may be due to the constant conflict, difficulty in resolving problems, and poor communication that can create a great amount of strain in relationships and lead to depressed mood.^44,45^ Depression can affect a person’s relationship with their partner and may cause emotional changes such as an increase in irritability, tiredness, and a lack of interest in socializing with their partner or activities they previously enjoyed together. It can be difficult to determine whether a relationship is contributing to emotional difficulties or if depression is causing problems within the relationship. However, in cases where a relationship is healthy, treating the depression may improve someone’s relationship with their partner as well as their own quality of life. Over time, frequent conflict can cause a breakdown in trust, communication, and emotional safety, all of which can fuel depression symptoms.^46,47^ Contrary to traditional notions, our study emphasizes that the middle ground of marital comfort, specifically the category of “somewhat comfortable,” emerges as a crucial predictor of depression. This deviation from the conventional understanding where extreme ends dictate outcomes underscores the complexity of the interplay between marital contentment and psychological health. Future research, inspired by these insights, should delve deeper into the mechanisms.

The use of injectable hormonal contraceptives is associated with an increased risk of depression compared to the use of oral hormonal contraceptives. This may be due to the synthetic form of progesterone, progestin, found in injectable contraceptives such as Depo-Provera. Progestin may increase levels of monoamine oxidase, which degrades serotonin concentrations and potentially leads to depression.^18^ Additionally, the action of progesterone metabolites on the γ-aminobutyric acid a receptor complex, which is the major inhibitory system in the human central nervous system, may play a role in the development of depression. Activation of the GABA system, which is involved in the pathogenesis of depression, is also a likely mechanism.^16^ This finding is supported by reports from the United States, Australia, and Germany, which have found an increased risk of depression among users of injectable hormonal contraceptives.^23,26,35^ However, it contradicts a study conducted in Saudi Arabia, which reported a higher risk of depression among oral contraceptive users compared to other types.^17^ The hormonal content difference of the contraceptives, which can vary across different brands, may contribute to this discrepancy.

The current study found a significant association between women with a BMI above 25 kg/m^2^ and depression. This is consistent with existing literature, which suggests that individuals who are overweight or obese are more prone to developing depression. This may be because obesity may involve dysregulation of the hypothalamic–pituitary–adrenal axis (HPA axis), and depression is known to be accompanied by dysregulation of the HPA axis. Obesity may lead to the development of depression through disruption of the HPA axis. In addition, obesity raises the risk of developing diabetes mellitus and increases insulin resistance, both of which can affect the brain and raise the likelihood of developing depression. It is possible to discuss psychological routes in addition to biological ones. Psychological anguish is exacerbated by being overweight and being perceived as overweight. According to reports from western nations, thinness is seen as the standard of beauty, and partially due to social acceptance and sociocultural factors, obesity may raise body dissatisfaction and deplete self-esteem, which are risk factors for depression.^48–50^ With increasing globalization and exposure to Western media and beauty standards, there is evidence that the Ethiopian perspective on body ideals has become more aligned with the Western emphasis on thinness as the epitome of attractiveness.^51,52^

### Strength and limitation

This study assessed severity of depression in addition to its prevalence and most important variables like social support, marital relationship, and BMI status. Women of different age-groups were included to fill the gap in knowledge in previous studies on the same subject. Depression was screened by BDI-II, which is a standard screening tool and increases the quality of assessment.

The study utilized a cross-sectional design, which can only establish associations between variables and cannot determine causality.

## Conclusion

Although contraceptive methods have given women greater control over their reproductive lives, mood-related side effects are less well documented and monitored than physical side effects. This study found a high and alarming prevalence of depressive symptoms among hormonal contraceptive users in Jimma town government health facilities.

Age category 18–25 years, poor social support, uncomfortable marital relationship, injectable type of hormonal contraceptive, and BMI >25 kg/m^2^ were statistically significant with depression.

Based on the findings above, the following recommendations are made to the Ministry of Health in collaboration with Jimma town administrative bodies and nongovernmental organizations that work on women’s health to expand mental health services in the town to prevent new incidences of depression and provide appropriate treatment for depressed women. Develop clear guidelines to assist practitioners working with women who are at risk of depression and who want to use hormonal contraceptives. Establish support groups and community networks to provide emotional and social support. Offer couples counseling and conflict resolution workshops to improve marital relationships and ensure access to domestic violence support services for those in abusive relationships. Implement weight management and healthy lifestyle programs, including nutritional counseling and physical activity initiatives. Develop age-specific mental health outreach programs focusing on the unique challenges faced by young women and provide accessible mental health resources, including hotlines and online support, tailored to young women. Exercise caution when prescribing injectable hormonal contraceptives to women.

In addition, based on the woman’s preferences, childbearing situation, and other medical issues, we advise considering nonhormonal contraceptives such as barrier methods, natural methods.

Finally, we suggest researches to conduct cohort studies to establish the cause-and-effect relationship between hormonal contraceptives and depression.

## Data Availability

The datasets used and/or analyzed during the current study are available within the article.

## Declarations

Details about the study and its benefits and potential risks were explained. Patients were recruited into the study after a written informed consent for those who were able to read and write and a fingerprint consent form for those who were unable to read and write were acquired. Fingerprint consent process was approved by Ethical Review Board of Jimma University. Data obtained during the study were kept confidential. Patients found to have severe depression during data collection were linked to the psychiatric department for further assessment and treatment. Furthermore, all methods were carried out in accordance with relevant guidelines and regulations.

Ethical approval was gained from the Institutional Ethical Review Board of Jimma University, Institute of Health (Registration No. 78/22). Then an official letter of cooperation was taken from the Jimma University Institute of Health Ethical Review Board and submitted to each health facilities. The study was approved by the Institutional Ethical Review Board of Jimma University, Institute of Health (Registration No. 78/22).

## Abbreviations Used

AOR: adjusted odds ratio
BDI: Beck Depression Inventory
BMI: body mass index
CI: confidence interval
ETB: Ethiopian Birr
GHQ-12: General Health Questionnaire 12
SCID: semi-structured clinical interview of diagnostic manuals for mental disorders
